# Tiptoeing on Metastatic Adenocarcinoma of the Lung

**DOI:** 10.1155/2019/9302861

**Published:** 2019-11-14

**Authors:** Arupreet Kochar, Harrison D. Winters, Li Zhang, Bin Zhang, Kamia Thakur, Rajesh Essrani

**Affiliations:** ^1^General Internal Medicine, Geisinger Medical Center, Danville, PA, USA; ^2^Hematology/Oncology, Geisinger Medical Center, Danville, PA, USA; ^3^Pathology and Laboratory Medicine, Geisinger Medical Center, Danville, PA, USA

## Abstract

Lung cancer remains the top cause of cancer-related death in the United States. Metastases to the distal phalanges are rare. This case illustrates an interesting presentation of metastatic adenocarcinoma of the lung that was mistaken for a benign condition of the toe.

## 1. Introduction

Metastases to the bone develop in 30% of all patients with cancer. While the majority of cancers that metastasize to the bone involve the spine, ribs, and pelvis, it has been reported that only 0.007%-0.3% metastases to the bone involve acrometastases [1].

Cancers of the colon and genitourinary tract are the most common primary cancers that have been reported to metastasize to the foot. While other primary cancers, such as adenocarcinoma of the lung, breast, prostate, and esophagus, have been described in case reports to metastasize to the foot, these lesions most commonly involve the talus, calcaneus, and metatarsal bones [2]. Acrometastases to the distal phalanges of the toe are among the rarest cases reported in the literature [3]. They are uncommonly the first presenting symptoms of the metastatic disease and can be mistaken for a benign condition, such as gout, bone cysts, or plantar fasciitis [2].

We present a rare case of adenocarcinoma of the lung with metastasis to the distal phalanx of the toe.

## 2. Case Presentation

**Figure 1:**
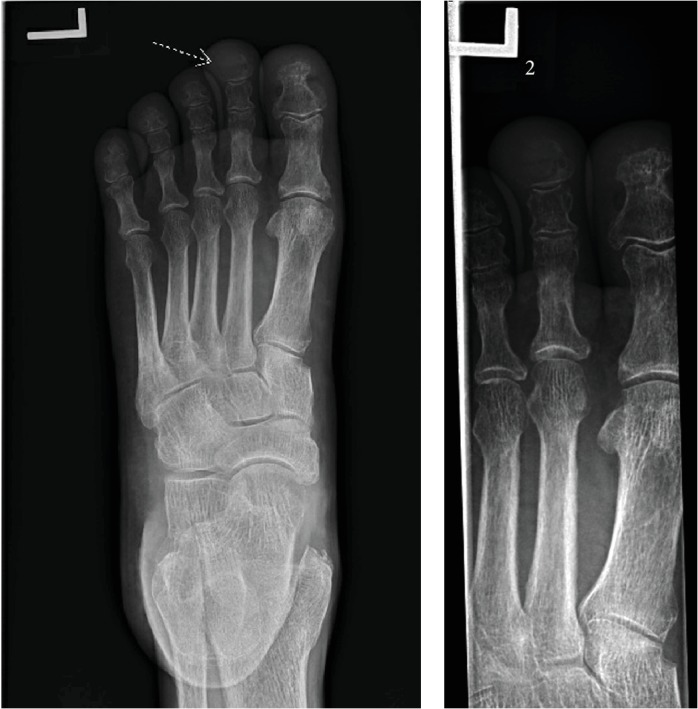
X-ray of left foot (AP lateral oblique views) demonstrating bony destructive lesion of the 2nd left toe.

**Figure 2:**
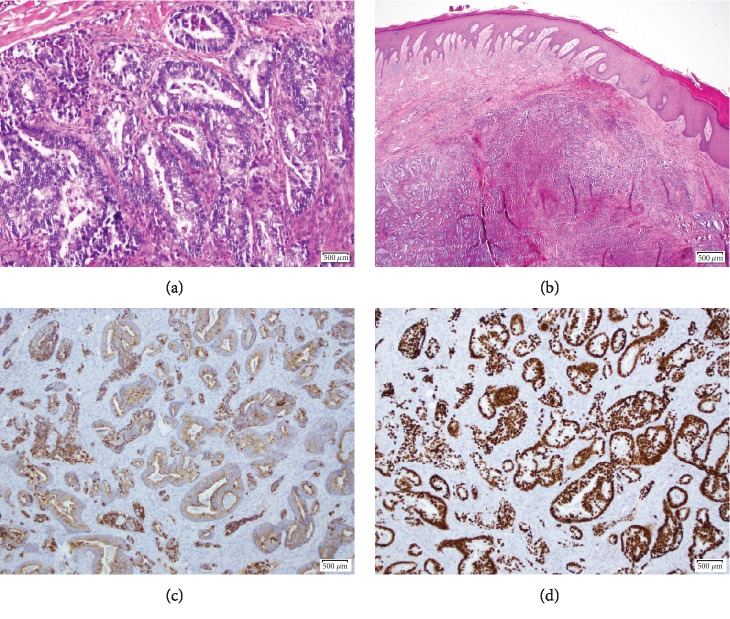
Lung adenocarcinoma metastasis to the left second toe. (a, b) The H&E section shows the dermal infiltration of adenocarcinoma; the tumor cells are arranged in a glandular growth pattern. No involvement of epidermis ((a) H&E 20x; (b) H&E 200x). (c, d) The immunohistochemical (IHC) stains reveal the tumor cells to be positive for CK7 ((c) 100x) and TTF-1 ((d) 100x), while negative for CK20, CDX-2, PAX-8, and NKX3.1 (data not shown). The previous lung fine needle aspiration slides were reviewed and found to be similar to the current specimen. Taken together, the morphology in combination with the immunoprofile are consistent with metastatic adenocarcinoma of the primary lung.

**Figure 3:**
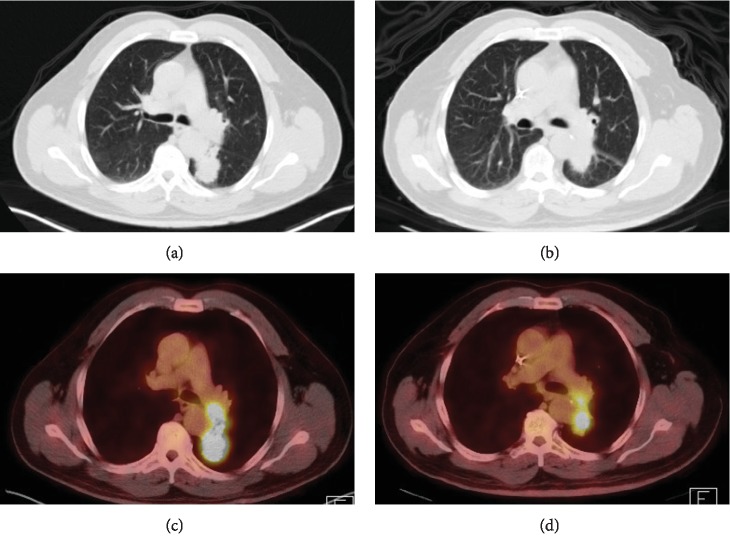
CT and fused PET/CT imaging (axial). (a, b) Staging CT and fused PET/CT of the base of the skull to midthigh showing a FDG avid mass in the left lower lobe of the lung with adjacent left hilar lymphadenopathy. Max SUV 14.83. (c, d) Restaging CT and fused PET/CT of the full body approx. 4 months after initiation of chemotherapy showing reduction in size of FDG avid mass in the left lower lobe of the lung with adjacent left hilar lymphadenopathy.

A 64-year-old male with a history of tobacco abuse and newly diagnosed adenocarcinoma of the lung presented to a podiatrist due to significant left second toe pain. He was clinically diagnosed with gout and treated with meloxicam. Three days later he returned to the podiatrist due to worsening symptoms and was diagnosed with paronychia and treated with clindamycin. His condition continued to deteriorate and prompted an emergency room visit at the hospital. On physical exam, his left second toe was erythematous, swollen, and tender to palpation. There was a decreased range of motion in his left foot due to pain. Dorsalis pedis pulses were +2/4 bilaterally. White blood cell count, ESR, and CRP were mildly elevated. ANA, uric acid level, and CMP were unremarkable. An X-ray of the foot revealed bone destruction involving the left second distal phalanx (Figure 1). He underwent a disarticulation and partial amputation of middle phalanx with pathology that was consistent with metastatic adenocarcinoma of the lung (Figure 2). Subsequent MRI of the brain confirmed stage IV adenocarcinoma of the lung. He was treated with stereotactic radiation to the brain and chemotherapy, consisting of carboplatin, pemetrexed, and pembrolizumab. Three months after radiation, follow-up MRI of the brain showed complete resolution of one lesion affecting the frontal lobe and an interval decrease in the size of the other two brain metastases. Four months after initiation of chemotherapy, there was found to be an interval decrease in size and FDG activity of the left lower lobe lung mass on PET imaging (Figure 3). The patient is responding well to treatment.

## 3. Discussion

This case illustrates a unique presentation of metastatic adenocarcinoma to the distal phalanx. While it is a rare phenomenon, suspicion for bony metastasis should be raised in any patient with relevant risk factors for malignancy or newly diagnosed carcinoma. It is interesting to note that using a PET scan to help properly stage this lung cancer case missed any evidence of a distant metastasis to the foot, since these scans are conducted from the skull to midthigh. Therefore, this case highlights the importance of conducting a thorough medical history in order to quickly identify signs and symptoms of metastatic disease that may appear similar to infection or an inflammatory process. Quickly recognizing signs of metastatic disease is essential to maximize the quality of life with appropriate treatment, as well as for the overall staging, management, and outcome of oncologic diseases.

## 4. Conclusions

- Non-small cell lung cancers most commonly metastasize to solid organs, including the liver and brain, as well as the bones, and lymph nodes
- Metastases to the distal phalanges are rare, and in this case, metastatic adenocarcinoma of the lung was mistaken for a benign condition of the foot
- Staging of lung cancer for metastases is completed by imaging the body from the skull to midthigh with PET imaging, which in this case missed distant foot metastasis
- Whole body PET scan is not done routinely for staging of lung cancer
